# Age-related changes in B cell metabolism

**DOI:** 10.18632/aging.102058

**Published:** 2019-07-08

**Authors:** Raj K. Kurupati, Larissa H. Haut, Kenneth E. Schmader, Hildegund CJ. Ertl

**Affiliations:** 1 The Wistar Institute, Philadelphia, PA 19104, USA; 2 Division of Geriatrics, Department of Medicine, Duke University Medical Center, Durham, NC 27710, USA; 3 Geriatric Research, Education, and Clinical Center, Durham VA Medical Center, Durham, NC 27705, USA

**Keywords:** vaccination, antibody responses, B cell metabolism

## Abstract

Antibody responses to vaccinations or infections decline upon aging. In this study we tested if metabolic changes in B cells may contribute to attenuation of responses to influenza vaccination in aged humans. Our data show that aging affects mitochondrial functions in B cells leading to increases in mitochondrial reactive oxygen species (MROS) and mitochondrial mass (MM) in some aged B cell subsets and decreases in expression levels of Sirtuin 1 (SIRT1), Forkhead box protein (FOX)O1 and carnitine palmitoyltransferase 1 (CPT-1). Seahorse analyses showed minor defects in glycolysis in the aged B cells after activation but a strong reduction in oxidative phosphorylation. The analyses of the transcriptome revealed further pronounced defects in one-carbon metabolism, a pathway that is essential for amino acid and nucleotide metabolism. Overall our data support the notion that the declining ability of aged B cells to increase their metabolism following activation contributes to the weakened antibody responses of the elderly.

## INTRODUCTION

Aged as compared to younger humans show reduced responsiveness to annual influenza vaccination, rendering them more susceptible to life-threatening infections [1–3]. Protection against influenza virus is predominantly mediated by neutralizing antibodies [4], which are produced upon influenza vaccination either by recalled memory B cells that had been primed by previous vaccinations or infections or by naïve B cells stimulated by new antigenic determinants of the annual vaccines. The aged typically have lower numbers of naïve B cells due to reduced output from the bone marrow [5]. Aged B cells also show defects in class-switching [6], which reduces their ability to develop antibodies with varied functions. Furthermore, the aged show diminished diversity in their B cell repertoire [7]. Additional defects in CD4^+^ T helper cells, which are essential to drive B cell stimulation, contribute to the attenuated antibody responses to influenza vaccination in the aged [8].

Following stimulation, T and B cells undergo metabolic changes to allow for increased energy and biomass production required for cell proliferation and production of effector molecules [9–11]. B cells that transition from a quiescent to an activated stage show increased uptake of glucose and enhanced energy production through glycolysis. They also display increased conversion of pyruvate, the end product of glycolysis, into acetyl-CoA [12], which can be used in the tricarboxylic acid (TCA) cycle for mitochondrial energy production or lipid synthesis to support production of the dividing cells’ membranes.

Aging is accompanied by metabolic changes such as enhanced resistance to insulin [13, 14], decline in mitochondrial functions [15, 16], changes in nutrient uptake [17] and circadian shifts [18, 19]. Here we tested if differences in B cell metabolism could contribute to reduced antibody responses to influenza vaccination in the aged. Data were generated using staining and flow cytometric analysis for metabolic markers, Seahorse analyses to assess use of glycolysis and the TCA cycle upon *in vitro* stimulation of naïve B cells, and comparisons of transcript expression levels for relevant enzymes of different metabolic pathways. As expected, our results show that the aged develop lower influenza virus-specific antibody titers upon vaccination with the trivalent inactivated influenza vaccine (TIV) and this is linked to differential expression of metabolic markers in different B cell subsets. In addition, aged naïve B cells stimulated *in vitro* show reduced mitochondrial energy production and exhibit reduced transcripts for key enzymes of one-carbon metabolism, both of which could contribute to weakened antibody responses.

## RESULTS

### Patient characteristics and antibody responses

**Figure 1:**
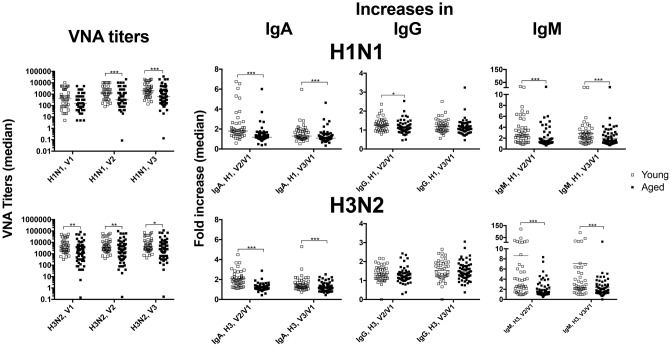
**Antibody responses.** Sera from younger (open squares) and older (closed squares) individuals were tested for VNAs and antibodies of different serotypes specific for H1N1 (upper graphs) or H3N2 viruses (lower graphs). The graphs show absolute values for VNA titers and fold increase over baseline (visit 1 [V1]) for IgA, IgG and IgM by dividing amounts of antibody in μg/ml (extrapolated from using standards for each isotype) after vaccination by those obtained at baseline. Graphs show data for individual samples with medians. Lines with star above indicate significant differences by Mann-Whitney. * p-value between <0.05-0.01, ** p-value between <0.01 and 0.001, *** p-value between <0.001 and 0.0001, **** p-value <0.0001.

We tested antibody responses to influenza A viruses present in the annual TIV vaccines in 43 younger individuals between 30 to 40 years of age (median age: 33) and 65 elderly persons between the ages of 65 to 89 (median age: 77) before and after their vaccination with TIV during fall of 2013 and 2015. Within the younger cohort 67% were female and 93% were Caucasians. Distributions in gender and race were similar in the elderly with 72% females and 92% Caucasians. In both cohorts, 95% of individuals reported previous influenza vaccinations; 40% of the younger and 48% of the aged had received the vaccines each year for the 5 years prior to 2013 or 2015. Blood was collected at visit (V) 1 just prior to vaccination, and on days 7 (V2) and 14 or 28 (V3) after vaccination. Sera were tested for virus neutralizing antibodies (VNAs) and IgA, IgG and IgM to the two influenza A viruses present in the vaccine. As expected and reported previously by us and others [1, 3], antibody titers to H1N1 and H3N2 at baseline as well as increases in titers following vaccination but for H3N2-specific IgG were lower in elderly than younger individuals (Figure 1).

### Expression of metabolic marker on/in aged and younger B cells

Changes in metabolism are one of the hallmarks of aging [20, 21]. Following activation, lymphocytes have to increase energy and biomass production to allow for their proliferation and production of effector molecules [22]. To assess if the lower antibody responses of the aged could be linked to age-related metabolic changes in different B cell subsets, blood-derived lymphocytes collected at baseline from subsets of our cohorts were stained with B cell subset defining antibodies [1] and panels of antibodies to different metabolic markers. We tested naive, non-switched and switched memory B cells as well as antibody secreting cells (ASCs). The gating strategy for the different subsets is shown in Supplementary Figure 1. Briefly, lymphoid single cells were gated on live cells, which were then gated on CD3^-^CD14^-^ cells. CD19 was used to identify B cells. CD19^+^ cells were gated onto IgD^-^ and IgD^+^ cells. IgD^+^ cells were gated onto CD27 and CD38. Mature naïve cells were identified as IgD^+^CD27^-^CD38^-^ cells while IgD^+^CD27^+^CD38^-^ cells were classified as unswitched memory B cells. IgD^-^ cells were separated into CD20^+^ and CD20^-^ cells. The former were gated onto CD27 and CD38 to identify switched memory B cells (CD38^+^CD27^-^). CD20^-^ cells were also gated onto CD27 and CD38 to identify CD27^+^CD38^+^ ASCs.

**Figure 2:**
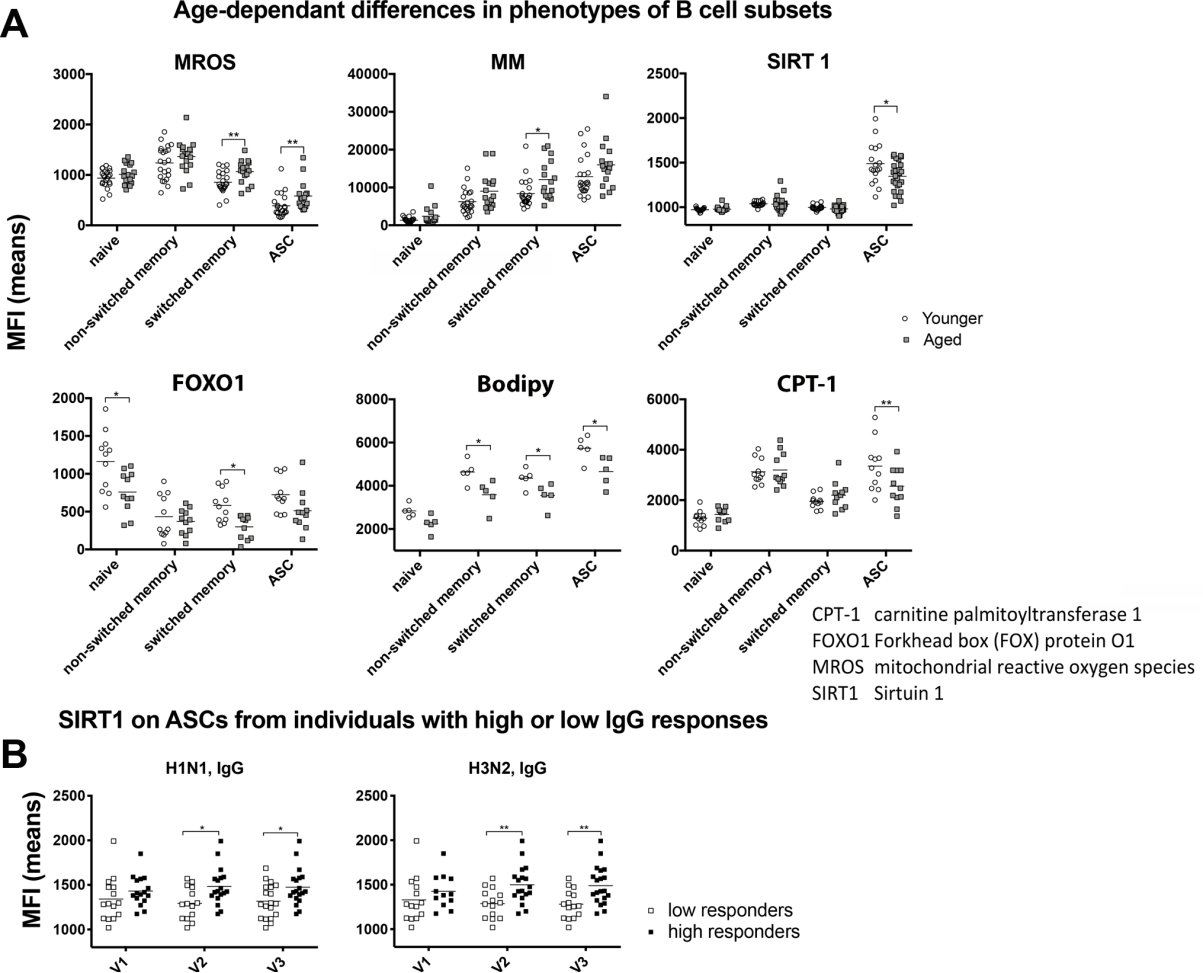
**Metabolic phenotypes of B cells.** (**A**) Graphs show mean fluorescent intensity (MFI) of stains for the indicated markers in or on different B cell subsets, i.e., naïve B cells (IgD^+^CD19^+^CD27^-^CD38^-^), unswitched memory B cells (IgD^+^CD19^+^CD27^+^CD38^-^), switched memory B cells (IgD^-^CD20^+^CD19^+^CD38^+^CD27^-^) and ASCs (IgD^-^CD20^-^CD19^+^CD38^+^CD27^+^) of younger (open circles) or aged (grey squares) individuals. Graphs show results for individual samples with means. Statistical difference indicated with lines and stars above as in legend to Figure 1 were calculated with multiple type 1 error corrected t-tests. (**B**) Samples were separated into those with high antibody responses (~1/3 of samples) and low antibody responses (~1/3 of samples). To this end samples were sorted according to magnitude of increases of the different antibody responses (VNAs, IgA, IgM and IgG titer increases) after vaccination as compared to baseline. The top and bottom samples (~1/3 of all samples each) were selected and analyzed. Significant differences were seen for the MFI for the SIRT1 stain in ASCs in high versus low IgG responders to H1N1 and H3N2. Lines with stars above indicate significant differences as described in legend to Figure 1. Abbreviations: CPT-1: carnitine palmitoyltransferase 1; FOXO1: Forkhead box (FOX) protein O1; MROS: mitochondrial reactive oxygen species; SIRT1: Sirtuin 1.

B cell subsets were tested for cellular and mitochondrial reactive oxygen species (C/MROS); mitochondrial membrane potential (MMP); mitochondrial mass (MM); phosphorylated protein kinase B (pAKT), a key enzyme that controls cell proliferation, survival and glucose (Glc) uptake [23]; solute carrier family 2 (Slc2, also termed Glut1), the main transporter used by lymphocytes for Glc uptake [24]; FOXO1, which regulates metabolic homeostasis in response to oxidative stress [25]; and SIRT1, an NAD-dependent protein deacetylase involved in cell cycling, DNA damage responses, metabolism and apoptosis [26]. A smaller set of samples from younger and aged individuals (10 each) enrolled in year 2014 were tested for levels of expression of SIRT 3 [27], peroxisome proliferator-activated receptor (PPAR)-α, the master regulator of fatty acid metabolism [28] and carnitine palmitoyltransferase 1 (CPT-1), a key enzyme of mitochondrial fatty acid beta-oxidation (FAO) [29]. We also tested for uptake of Bodipy FL C16 to assess differences in transport of a fatty acid [30]. As shown in Figure 2A several of these markers, i.e., MROS, MM, SIRT1, FOXO1, fatty acid uptake and CPT-1, showed statistically significant differences by type 1 error-corrected multiple t-tests in their expression in B cell subsets of the younger and elderly study participants. Switched memory B cells (IgD^-^CD20^+^CD19^+^CD38^+^CD27^-^) and ASCs (IgD^-^CD20^-^CD19^+^CD38^+^CD27^+^) of the elderly had higher levels of MROS, aged memory B cells had increased MM while ASCs from younger individuals had higher expression of SIRT1. FOXO1 was higher on younger naïve (IgD^+^CD19^+^CD27^-^CD38^-^) and memory B cells. Younger B cells, but for naïve B cells, showed higher uptake of Bodipy FL C16. CPT-1 expression was higher in younger than aged ASCs although in naïve and memory B cells there was a trend towards higher expression in those from the aged. For some of the markers there were also pronounced differences between B cell subsets. MROS was lowest in ASCs, while expression levels of MM, SIRT1 and Cpt-1, and fatty acid uptake were highest in or by ASCs. In contrast, FOXO1 was highest in naïve B cells and declined upon their activation. The other metabolic markers were expressed at comparable levels in or on B cells from the different subsets from younger or aged individuals (Supplementary Figure 2) although expression levels differed between subsets. pAKT, Glut1 and MMP were highest in/on ASCs while expression of SIRT3 and PPAR-α were elevated in non-switched memory B cells (IgD^+^CD19^+^CD27^+^CD38^-^).

To assess if differential expression levels of metabolic markers in ASCs was linked to influenza virus-specific antibody responses, we compared expression in individuals with the lowest and highest antibody responses regardless of age. As shown in Figure 2B significant differences were observed for SIRT1, which was significantly higher in ASCs from individuals that developed more potent IgG responses to H1N1 and H3N2.

### Pathways of energy production by aged and younger B cells

**Figure 3:**
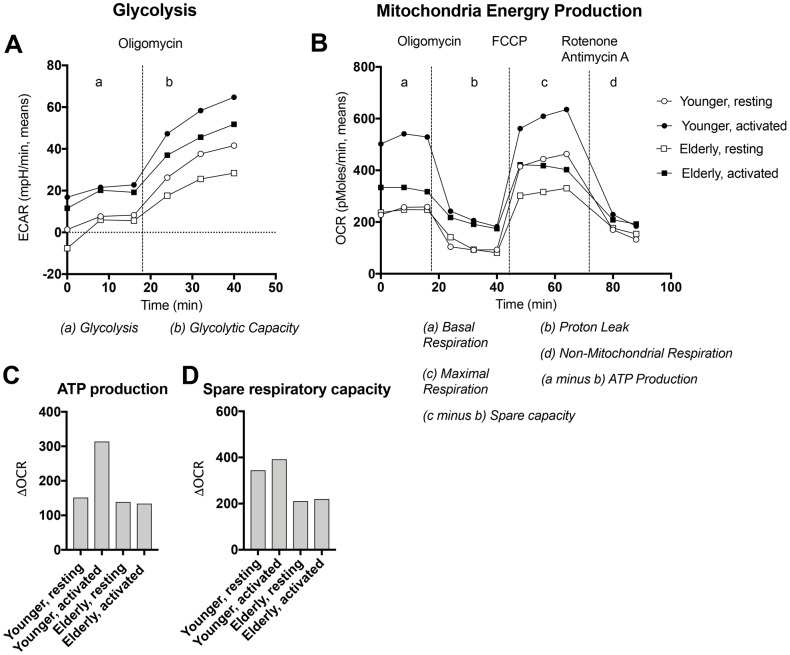
**Energy production by naïve B cells undergoing activation.** Naïve B cells isolated from PBMCs of younger (circles) or aged (squares) individuals were cultured for 24 hours with (closed symbols) or without (open symbols) polyclonal activators and then tested by Seahorse for glycolysis (**A**) or respiration (**B**). After 3 measurements at baseline oligomycin was added to determine glycolytic capacity and proton leak. After 3 measurements carbonyl cyanide-4-phenylhydrazone (FCCP) was added to maximal respiration. Thereafter Rotenone and Antimycin A were added to measure non-mitochondrial respiration. Data for oxygen consumption rates (OCR) were used to calculate ATP production as basal respiration minus proton leak (**C**) and spare respiratory capacity as maximal respiration minus non-mitochondrial respiration (**D**).

To further assess age-related changes in energy production, we collected blood from additional younger and aged individuals and isolated naïve B cells by negative selection. Cells were cultured for 24 hours either without stimulation or with polyclonal activators, i.e., *S. Aureus* capsid, PokeWeed Mitogen extract, a CpG oligonucleotide (ODN 2006), human-IL-4 and anti-CD40 antibody. Extracellular acidification rates (ECAR), a measure for lactate production by glycolysis, and oxygen consumption rates (OCR) to test for mitochondrial energy production were then analyzed by Seahorse [31]. After several baseline measurements, oligomycin (OM) was added to determine glycolytic capacity and to stop ATP production and measure proton leaks. Thereafter carbonyl cyanide-4-phenylhydrazone (FCCP) was added to determine maximal respiration followed by addition of Rotenone and Antimycin D to assess non-mitochondrial respiration. As shown in Figure 3A, at baseline young and aged naïve B cells that were resting or stimulated, showed similar levels of lactate production indicating comparable rates of glycolysis. For both populations, ECAR were higher for polyclonally activated than resting B cells. After addition of oligomycin, ECAR increased more pronounced in cultures with younger than aged B cells.

Differences were more striking for OCR (Figure 3B). Already at baseline, aged B cells showed reduced respiration compared to those from younger individuals as we described previously for mouse B cells [32]. Younger B cells consumed more O_2_ after stimulation, while resting and activated aged B cells showed no differences in OCR. As expected, OCR decreased after addition of OM showing more pronounced proton leaks in younger than older B cells. The difference between OCR at baseline and upon addition of oligomycin, was used to calculate ATP production, which increased in the younger but not the aged B cells after stimulation. OCR increased after addition of FCCP. The spare respiratory capacity calculated as OCR after FCCP addition minus OCR in presence of OM, was higher in younger than aged resting or stimulated B cells.

Overall these results indicate mild deficiencies in glycolysis in the aged with a reduced glycolytic capacity and more pronounced defects in mitochondrial energy production.

### Levels of transcript encoding factors involved in metabolic pathways in aged and younger B cells

**Figure 4:**
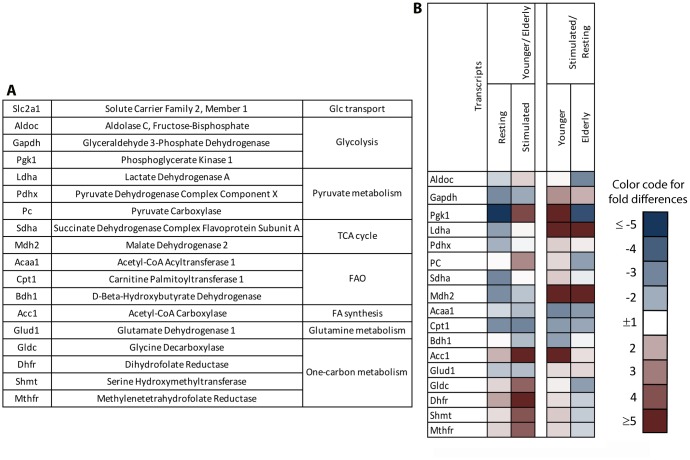
**Gene expression profiles of naïve B cells undergoing stimulation.** Naïve B cells isolated from PBMCs of younger or aged individuals were cultured for 24 hours with (stimulated) or without (resting) polyclonal activators. cDNA was generated and probed with primers to the indicated factors (**A**) by comparative (c)PCR. (**B**) The heatmap to the left shows age-related differences in levels of transcripts between resting or stimulated B cells comparing younger to elderly individuals. The heatmap to the right shows activation induced changes in younger and aged B cells. Blue shows reduced levels and red shows enhanced levels of transcripts in the younger compared to aged B cells (left) or in stimulated compared to resting B cells (right). Color intensities indicate magnitude of differences as shown in the legend.

To further pinpoint potential metabolic changes upon aging, we isolated naïve B cells from blood of younger and aged individuals and cultured them without further activation (resting) or upon stimulation with polyclonal activators. RNA was isolated 24 hours later and upon reverse transcription, probed with primers for transcripts encoding different enzymes and factors involved in key metabolic pathways (Figure 4A) in a comparative PCR. Specifically, we compared resting B cells, i.e., B cells that had been cultured without activators of younger individuals to those of the aged, and conducted the same comparison for B cells that had been stimulated *in vitro* to assess age-related differences. In addition, we compared the transcriptional profiles of activated to resting B cells within the two age groups to determine differences in activation induced changes. As shown in Figure 4B, in the comparison of younger to aged B cells, resting younger B cells had lower levels of transcripts for enzymes of glycolysis, pyruvate metabolism, the TCA cycle, fatty acid and glutamine catabolism while transcripts for enzymes involved in fatty acid synthesis or one-carbon metabolism were slightly higher. Upon stimulation aged B cells continued to express slightly higher levels of enzymes involved in the TCA cycle, fatty acid and glutamine catabolism. Levels of transcripts for enzymes of glycolysis or pyruvate metabolism in stimulated aged B cells were in part similar to those of younger B cells although transcripts for phosphoglycerate kinase 1, an ATP generating reversible enzyme of glycolysis, and pyruvate carboxylase, which converts pyruvate to oxaloacetate, a TCA intermediate involved in several anabolic pathway were higher in younger B cells. The most striking differences were seen in transcripts for Acetyl-CoA carbocylase (ACC1), an enzyme that is needed for fatty acid biosynthesis and enzymes of one-carbon metabolism; all of them were markedly higher in stimulated younger than aged B cells. Expression profiles of stimulated versus resting B cells showed, in both younger and aged B cells, marked increases in levels of lactate dehydrogenase A and malate dehydrogenase 2, slight increases in glutamate dehydrogenase 1. Decreases in transcripts for enzymes of fatty acid catabolism upon stimulation indicated increased activity of glycolysis, glutaminolysis and the TCA cycle, and reduced fatty acid beta oxidation. The main age-related differences between stimulation-induced transcriptional changes were observed for aldolase C, phosphoglycerate kinase 1, pyruvate carboxylase and transcripts of enzymes involved in fatty acid synthesis and one-carbon metabolism: increases were observed after stimulation of younger B cells but declined or did not change in the aged. Activation-induced increases in other transcripts, such as those for pyruvate dehydrogenase and succinate dehydrogenase, were less pronounced in aged than younger B cells upon stimulation.

## DISCUSSION

During aging the immune system gradually deteriorates, increasing the elderly’s susceptibility to infections while decreasing their responsiveness to vaccines. Declines of immune functions may relate to changes in metabolism. Foremost, resting T and B cells use the highly efficient TCA cycle for energy production. Stimulation of both lymphocyte subsets increases uptake of Glc to feed energy production by glycolysis [33]. T cells convert most of the Glc via pyruvate to lactate, while B cells with their higher activity of pyruvate dehydrogenase [12] metabolize more of the pyruvate to acetyl CoA, which either enters the TCA cycle or provides building blocks for lipogenesis. B cells also increase uptake and catabolism of amino acids [34, 35] but with the exception of peritoneal B1 cells, B cells have thus far not been reported to enhance use of fatty acids for energy production [36]. Aged lymphocytes may have difficulties to adjust their metabolism to fuel increased proliferation and production of effector molecules and this could be one of the reasons for the attenuated immune responses of the aged. Aged T cells accumulate mitochondrial defects [37], have decreased mitochondrial biogenesis [38] and increased levels of MROS [39]. They were shown to enhance use of glycolysis, which was linked to reduced expression of SIRT1 [40].

Results here confirm some of these finding for aged B cells. As has been described previously [1–3], as in our study, B cells from the elderly were less functional as antibody responses to TIV were weakened. In agreement with previous results obtained with mouse B cells [32], aged human B cells showed reduced mitochondrial energy production while glycolysis was less affected in aged resting or stimulated B cells. Aged B cells showed additional mitochondrial defects such as increases in MROS [41] and MM [42] as has been described previously for other cell types. It has been reported that mitochondrial functions play a role in B cell fate decisions [43], specifically MROS can maintain functions of Bach2, which in turn through inhibition of Blimp-1 promotes class switching and blocks differentiation of B cells into plasma cells. Increased levels of MROS in aged B cells could thus explain why in our study class-switched influenza A virus-specific IgG responses were less affected in the elderly than IgM responses. This contrasts previous studies, which reported reduced class-switching in aged B cells due to impaired activity of activation-induced cytidine deaminase [44].

Our data show that ASCs had higher levels of SIRT1 than quiescent B cells although expression was significantly lower in aged ASCs. SIRT1 has a number of key functions [45, 46]. It is an NAD-dependent deacetylase, which can inactivate p53 and inhibit NF-κB regulated gene expression. It regulates expression of pyruvate dehydrogenase, which is essential for the use of Glc in mitochondrial energy production. In most tissues SIRT1 decreases glycolysis and increases fatty acid oxidation. Accordingly, ASCs showed the highest level of fatty acid uptake and had increased CPT-1 expression compared to naïve B cells and both were lower in the aged ASCs. The importance of SIRT1 expression in ASCs for high antibody responses is further underscored by our finding that independent of age individuals with high SIRT1 expression had significantly higher TIV-specific IgG responses compared to those with low SIRT1 levels. Other metabolic markers were expressed at comparable levels in younger and aged B cells such as Glut-1, which is compatible with our finding that glycolysis was only marginally affected in aged B cells.

*In vitro* studies designed to test for age-related transcriptional differences during early stimulation of naïve B cells showed that upon activation aged B cells showed marked reductions in transcripts for fatty acid synthesis and the one-carbon metabolism. One-carbon metabolism plays key roles in amino acid and nucleotide synthesis, DNA methylation and redox defenses [47]. It is thereby essential to allow for cell proliferation and epigenetic regulation of gene expression. Studies with mouse CD4^+^ T cells showed that increased activity of one-carbon metabolism is essential for the cells’ activation and survival [48]. Defects in one-carbon metabolism have been implicated in neurodegenerative and cardiovascular diseases and may contribute to the development of some types of cancers such as colon cancer [49–51]. The effect of aging on one-carbon metabolism has not yet been studied in detail but our data show that this pathway may not only decline in the aged but also respond less vigorously to increasing demands imposed by cell stimulation. Metformin, a commonly-used anti-diabetes drug impairs one-carbon metabolism [52] and as we reported reduces antibody responses to TIV in aged individuals again providing supporting evidence for the essential role of one-carbon metabolism on B cell functions [53].

In summary our study shows that in the aged defective antibody responses to immunization with TIV are in part reflective of age-related changes in mitochondrial energy production and one-carbon metabolism inviting additional studies on how such defects could be adjusted.

## MATERIALS AND METHODS

### Virus strains

The influenza A vaccine strains present in the 2013/14 and 2015/16 seasonal influenza vaccines, were obtained from the Center for Disease Control, Atlanta, Georgia. Influenza viruses were propagated in 10-day-old specific pathogen-free embryonated eggs as described [1]. Virus was purified from the allantoic fluids of the infected eggs by centrifugation over a 10-55% sucrose density gradient at 25,000rpm for 2 hrs. Purified viruses were serially diluted on Madin-Darby Canine Kidney (MDCK) cells and mean tissue culture infective doses (TCID_50_) were determined 3 days later by screening cells for cytopathic effects (CPE).

### Human subjects

Blood was collected after informed consent from community dwelling persons in the Durham-Raleigh-Chapel Hill area of North Carolina. Younger individuals were 30-40 years of age; older individuals were ≥ 65 years of age. From enrolled subjects, demographic data and medical history including vaccination to influenza and history of influenza or influenza-like diseases during the last 5 years were recorded. Subjects were bled and then vaccinated with TIV via the intramuscular route in the deltoid muscle. Subjects were bled again on days 7 and 14 or 28 following injection of TIV. Additional studies were conducted with blood freshly collected from younger or aged individuals by the Phlebotomy service at the Wistar Institute.

### Collection of blood and isolation of PBMCs and plasma

Blood was collected into heparinized tubes or into serum collection tubes. Blood collected in North Carolina was shipped overnight to Philadelphia, PA. Serum was isolated by centrifugation of tubes for 30 min at 2000 rpm. PBMCs were isolated by centrifugation of blood through Ficoll-Paque Plus (GE Healthcare Biosciences, Piscataway Township, NJ) for 30 minutes at 2000 rpm, with brake off, and at 50% acceleration. The PBMC layer at the Ficoll interface was collected and washed twice with Hank's Balanced Salt Solution (Gibco, Grand Island, NY) by centrifuging at 2000 rpm. The washed, pelleted cells were treated with 10 ml of red blood cell lysis buffer (eBioscience, San Diego, CA). Lysis was stopped by adding 5 ml of Roswell Park Memorial Institute (RPMI) 1640 medium supplemented with 10% fetal bovine serum (FBS). Cells were washed with Hank’s Salt (HBSS). Cells were resuspended in 5ml of Dulbecco’s modified Eagles medium (DMEM), live cells were counted using Trypan Blue as a diluent.

### Micro-neutralization assay

Two-fold serially diluted (1:20 to 1:10240), heat-inactivated sera were tested for neutralizing antibodies to influenza A virus strains by micro-neutralization assays. Equal volume of 100TCID_50_ per well of virus was added to the diluted serum in 96 well plates and incubated at 37°C. After 1hr, serum-virus mixtures were added to MDCK cells that had been washed twice with serum-free DMEM. The cells were incubated for 2 hrs at 37°C with 5% CO_2_. The cells were washed and re-incubated with DMEM supplemented with L -1-Tosylamide-2-phenylethyl chloromethyl ketone (TPCK) trypsin for 3 days. CPEs were scored under a microscope. Neutralization titers were defined as the dilution of the serum that resulted in 50% inhibition of CPE formation.

### ELISA

H1N1- and H3N2-specific binding antibody isotypes were measured by ELISA. Briefly, wells of Nunc Maxisorp™ plate were coated with 10μg/ml of influenza H1N1 or H3N2 virus or with isotype standards for IgA1, IgG and IgM (Athens Research & Technology, Inc., Georgia, USA) in bicarbonate buffer overnight at 4°C. The plates were blocked with 3% BSA in PBS and incubated for 2 hrs at room temperature with heat-inactivated sera at a dilution of 1/250. The plates were washed 4X with PBS containing 0.05% tween (PBST) and incubated for 1 hr at room temperature with alkaline phosphatase conjugated mouse anti-human IgA1 at 1:1000, IgG at 1:3000 and IgM at 1:1000 dilutions (SouthernBiotech, Alabama, USA). Plates were then washed 4X with PBST and developed using alkaline phosphatase substrate containing pNPP tablets (Sigma Aldrich, Missouri, USA) dissolved in DEA buffer. Adsorbance was read at 405nm. The adsorbance values were plotted against standard curves from each plate for every isotype. Antibody concentrations were determined and are expressed in μg/ml.

### B cell detection by flow cytometry

Each sample was treated with Human TruStain FcX Fc Receptor Blocking solution (BioLegend, San Diego, CA) for 30 minutes, washed with PBS at 1500 rpm for 5 minutes and then stained with fluorochrome-conjugated antibodies. Samples were stained first for their extracellular markers for 30 minutes at 4°C and after washing they were fixed and permeabilized with Cytofix/Cytoperm (BD Biosciences) for 30 minutes at 4°C. They were washed with Permwash (BD Biosciences) and stained with their specific panel of intracellular antibodies for 30 minutes at 4°C, washed with PBS and then resuspended in 150μl of fixative (BD Pharmingen). The following antibodies were used: Pacific Blue™ anti-human CD3 (Clone UCHT1, Biolegend), Pacific Blue™ anti-human CD14 (Clone M5E2, Biolegend; both as dump gates), Brilliant Violet 650™ anti-human CD19 (Clone HIB19, Biolegend), Brilliant Violet 570™ anti-human CD20 (Clone 2H7, Biolegend), Brilliant Violet 785™ anti-human CD27 (Clone O323, Biolegend), Brilliant Violet 711™ or PerCP/Cy5.5 anti-human CD38 (Clone HIT2, Biolegend), PerCP/Cy5.5 or PE/Cy7 anti-human IgD ((Clone IA6-2, Biolegend), APC/Cy7 anti-human IgM ((Clone MHM-88, Biolegend), BV605 Anti-Human IgG (Clone G18-145, BD Biosciences), Alexa Fluor® 488 Mouse anti-AKT (pS473) (Clone M89-61, BD Biosciences), Alexa Fluor® 700 Sirtuin 1/SIRT1 Antibody (Novus Biologicals), SIRT3 (Mouse IgG1 Clone # 850911, R&D Systems Inc., secondary anti IgG Alexa Fluor® 647), PE FOXO1 (Clone C29H4, Cell Signalling Technology), LIVE/DEAD™ Fixable Aqua (ThermoFisher Scientific), MM (MitoTracker™ Green FM, Em = FITC,ThermoFisher Scientific), CROS (CellROX™ Green, Em = FITC, ThermoFisher Scientific), MROS (MitoSOX™ Red, Em = PE, ThermoFisher Scientific), MMP (MitoTracker™ Orange CMTMRos, Em = PE, ThermoFisher Scientific), Alexa Fluor® 647 Glut1 (Clone EPR3915, ABCAM), Alexa Fluor® 488 CPT1A (Clone 8F6AE9, ABCAM), PE FOXO1 (Clone C29H4, Cell Signaling Technology, Inc.), Biotin PPAR alpha (Rabbit polyclonal, Abcam with secondary stain Streptavidin PE.CF594, BD Biosciences). Antibodies with identical or spectrally overlapping dyes were tested on separate samples. Fatty acid uptake was tested for by BODIPY™ FL C_16_(Em = Alexa Fluor® 488, ThermoFisher Scientific). The stained samples were tested in a LSRII flow cytometer (BD Biosciences, San Jose, CA) and analyzed by FlowJo.

### Isolation and stimulation of naïve B cells

Human naïve B cells were isolated with Naive B Cell Isolation Kit II, human (Miltenyi Biotec) according to the manufacturer’s instructions. The cells were stimulated for 24 hrs in RPMI medium with 10% FBS containing a cocktail of 1 μg/ml pokeweed mitogen (PWM) (Sigma-Aldrich, Saint Louis, MO), 1/10,000 dilution of heat-killed, formalin fixed *Staphylococcus aureus* Cowan I cells/PANSORBIN Cells (EMD Millipore, Billercia, MA), 3 μg/ml of oligo CpG ODN 2006 (synthesized at GenScript USA Inc., Piscataway, NJ) and 1 μg/ml of Anti-CD40 (BioXcell, West Lebanon, NH).

### Gene expression analysis

Naïve B cells were cultured *in vitro* with or without polyclonal activators. After 24 hours RNA was isolated from purified cells using RNeasy Mini kits (Qiagen) and RNA concentrations were determined using Nanodrop (Thermo Scientific). cDNAs were obtained by reverse transcription using the high capacity cDNA reverse transcription kit (Life Technologies). Relative qRT-PCR analyses were performed using 7500 Fast Real-Time PCR system (Life Technologies). Transcripts encoding 18S rRNA were used as internal controls. Vector NTI was used for primers design (Supplementary Table 1). Differences in transcript expression levels are visualized in heatmaps. Values were log transformed to show ratios of differences. Color scale was set as -5 (lower expression, deep blue) to 5 (higher expression, deep red).

### Extracellular flux analysis and FAO assay

OCR and ECAR for naïve B cells cultured with or without polyclonal activators were measured with XF24 and XF96 Extracellular Flux Analyzers (Seahorse Bioscience). Briefly after repeated measures of basal respiration and lactate production, 1μM OM was added to measure ATP leakage by OCR and glycolytic capacity by ECAR. 1.5μM FCCP was then added to measure maximal respiration by OCR followed by addition of 100nM Rotenone and 1μM Antimycin A to determine spare respiratory capacity.

### Statistical analyses

Results were analyzed by Mann Whitney, t-tests or 2-way ANOVA. P-values were corrected for multiple testing using the Holm-Sidak procedure. Results with corrected p-values ≤0.05 were considered significant.

## Supplementary Material

Supplementary Figures

Supplementary Table 1
