# Supplementary material for: Age-related changes in B cell metabolism
Source: Aging (Albany NY). 2019 Jul 8;11(13):4367–81. doi: 10.18632/aging.102058 (PMC6660053; doi:10.18632/aging.102058)
Supplement: Supplementary Figures [file aging-11-102058-s002.pdf]

SUPPLEMENTARY FIGURES

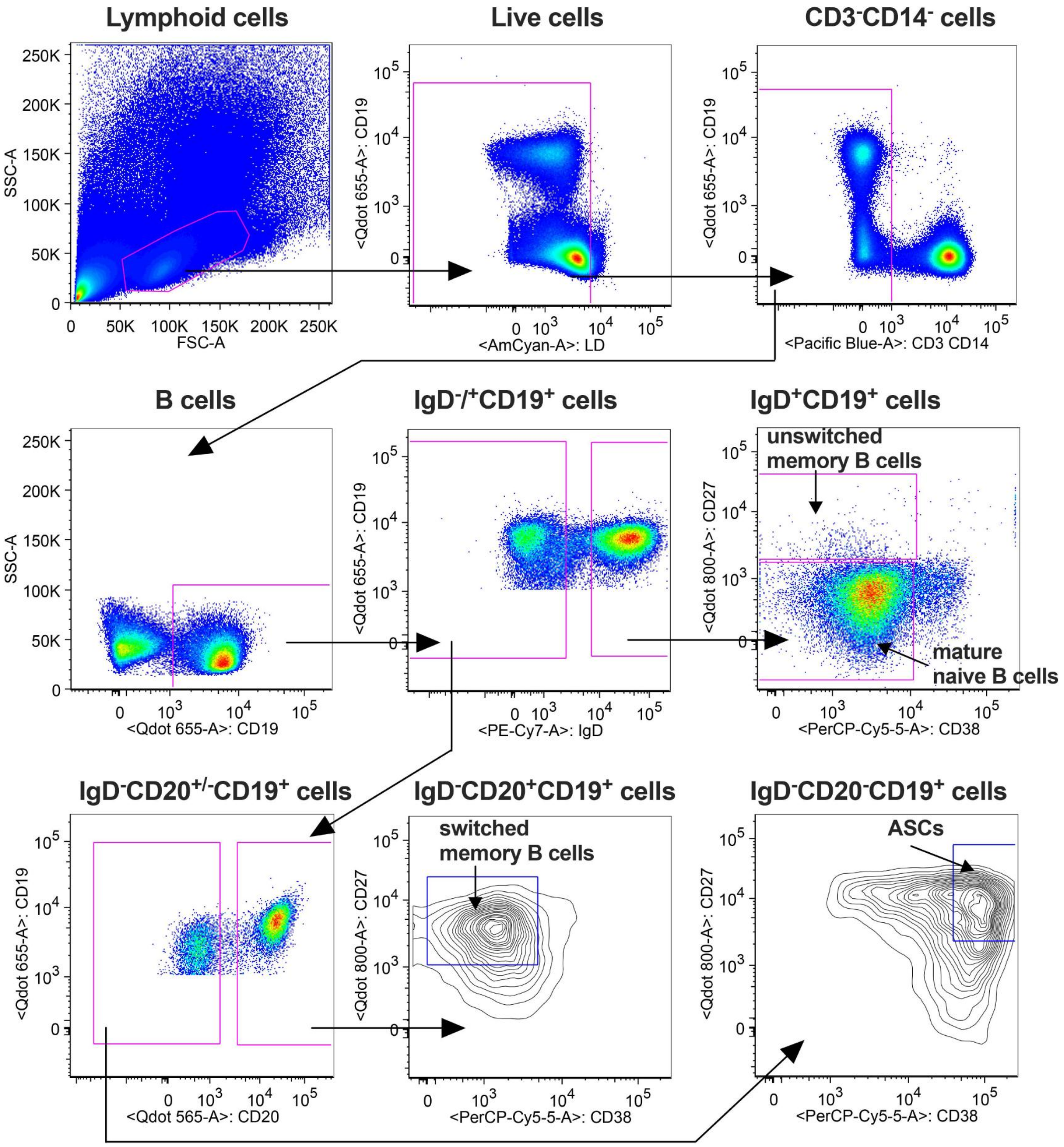

Supplementary Figure 1. Gating strategy for B cell subsets. PBMCs were stained with antibodies to CD3 and CD14 both labeled with Pacific Blue, a live cell stain, antibodies to CD19 (Qdot 655-A), IgD (PE-Cy7), CD27 (Qdot 800-A), CD38 (PerCP-Cy5-5-A) and CD20 (Qdot 565-A). Cells were first gate on lymphoid cells, then on singlets (not shown), live cells, $CD3^-CD14^-$ cells and $CD19^+$ B cells. B cells were gated onto IgD^+ cells to identify $CD27^-CD38^-$ mature naïve B cells and $CD27^+CD38^-$ unswitched memory B cells, IgD^- cells were used to identify $CD20^+$ and $CD20^-$ cells. $CD20^+$ cells were used to identify $CD27^+CD38^-$ switched memory B cells and $CD20^-$ cells were used to identify $CD27^+CD38^+$ ASCs. The sample was collected at v1 from an aged individual.

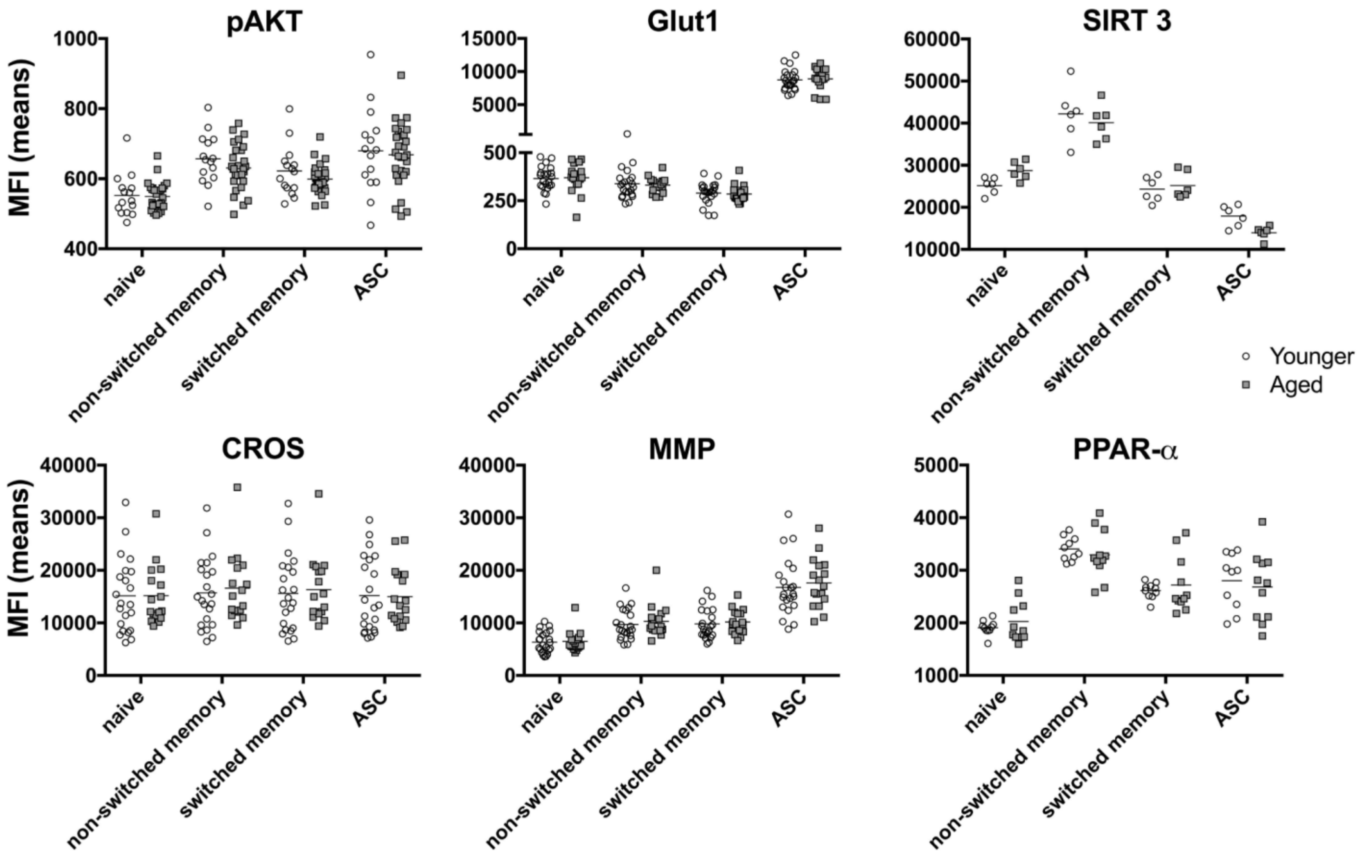

ASC antibody secreting cells
CROS cellular reactive oxygen species
Glut1 glucose transporter 1
MMP mitochondrial membrane potential

pAKT phosphorylated protein kinase B
PPARα peroxisome proliferator-activated receptor α
SIRT1 Sirtuin 1

Supplementary Figure 2. Metabolic phenotypes of B cells. Graphs show mean fluorescent intensity (MFI) of stains for the indicated markers in or on different B cell subsets, i.e., naïve B cells ($IgD^{+}CD19^{+}CD27^{-}CD38^{-}$), unswitched memory B cells ($IgD^{+}CD19^{+}CD27^{+}CD38^{-}$), switched memory B cells ($IgD^{-}CD20^{+}CD19^{+}CD38^{+}CD27^{-}$) and ASCs ($IgD^{-}CD20^{-}CD19^{+}CD38^{+}CD27^{+}$) of younger (open circles) or aged (grey squares) individuals. Graphs show results for individual samples with means.
