# Supplementary material for: Age-related changes in B cell metabolism
Source: Aging (Albany NY). 2019 Jul 8;11(13):4367–81. doi: 10.18632/aging.102058 (PMC6660053; doi:10.18632/aging.102058)
Supplement: Supplementary Table 1 [file aging-11-102058-s001.pdf]

SUPPLEMENTARY TABLE

Supplementary Table 1. Differences in transcript expression levels visualized in heatmaps.

Symbol	Forward	Reverse	GenBank Accession
Slc2a1	TGGATCCCAGCAGCAAGAAGG	TGAAGCGGCCCAGGATCAG	
Aldoc	TGCTGAGCAGAAGAAGGAGTTGTC	TCTCCACCCCAATTTGGCTC	NM_005165.2
Gapdh	TCCGGGAAACTGTGGCGTG	TCCCGTTCAGCTCAGGGATG	NM_002046.5
Pgk1	ATGTCGCTTTCCAACAAGCTG	TGGCTCCATTGTCCAAGCAG	NM_000291.3
Ldha	TTGACCTACGTGGCTTGGAAG	GGTAACGGAATCGGGCTGAAT	NM_001165415
Pdhx	GAGTGGTTGATGACGAACTGG	GGCAAGTCGGATAGGATTCTCTA	NM_003477
Pc	ACAGAGGTGAGATTGCCATCC	CACTGCATCTACGTTGTTCTCC	NM_001040716.1
Sdha	CAGCATGTGTTACCAAGCTGT	GGTGTCGTAGAAATGCCACCT	NM_004168
Mdh2	GCCATGATCTGCGTCATTGC	CCGAAGATTTTGTTGGGGTTGT	NM_005918
Acaa1	TCCGACGTGGTGGTGGTGC	TCCCAGCTGCTCAGGCTTTAG	NR_024024.1
Cpt1	TCCAGTTGGCTTATCGTGGTG	TCCAGAGTCCGATTGATTTTTGC	NM_001876
Bdh1	GACAGCCTAAACAGTGACCGA	GAGCGGACAATCTCCACCA	NM_203315
Acc1	AGTGTGGGCTGGCTGGGGTC	ATCCCCCAAAGCCCACATGG	NM_198836.2
Glud1	TTCAAGATGGTGGAGGGCTTC	TCCGCTTCTGCTCCTCGCTC	NM_005271.3
Gldc	CCAGACACGACGACTTCGC	CAATTCATCAATGCTCGCCAG	NM_000170.2
Dhfr	GAATCACCCAGGCCATCTTA	GCCTTTCTCCTCCTGGACAT	NM_000791.3
Shmt	CAATGACGATGCCAGTCAA	GAGGGGTTGTGCCAGCA	NM_148918.2
Mthfr	CTGCTGCACCAGAGTGAAAG	TATGGCCCTTGGACCTACTG	NM_005957.4
